# Sox9 downregulation in non-obstructive azoospermia by UTF1 and mediator role of POU5F1

**DOI:** 10.1186/s13104-024-06711-0

**Published:** 2024-03-14

**Authors:** Mehdi Mehdinezhad Roshan, Hossein Azizi, Mohammadreza Ashtari Majelan, Amirreza Niazi Tabar

**Affiliations:** 1 Faculty of Biotechnology, Amol University of Special Modern Technologies, 49767 Amol, P.O. Box: 49767, Iran; 2 Department of Biology and Anatomical Sciences, School of Medicine, Shahid Beheshti University of Medical Sciences, Tehran, Iran; 3 Department of Biology and Anatomical Sciences, Qazvin University of Medical Science, Qazvin, Iran

**Keywords:** Testis, Sertoli cells, Immunohistochemistry, Non-obstructive azoospermia

## Abstract

**Background:**

Spermatogenesis is the process of producing mature sperm from Spermatogonial stem cells (SSCs) and this process requires a complex cooperation of different types of somatic and germ cells. Undifferentiated spermatogonia initiate the spermatogenesis and Sertoli cells as the only somatic cells inside of the seminiferous tubule play a key role in providing chemical and physical requirements for normal spermatogenesis, here, we investigated the dysfunction of these cells in non-obstructive azoospermia.

**Material and method:**

In this study, we analyzed the expression of sox9 and UTF1 in the non-obstructive human testis by immunohistochemistry. Moreover, we used the KEGG pathway and bioinformatics analysis to reveal the connection between our object genes and protein.

**Results:**

The immunohistochemistry analysis of the non-obstructive human seminiferous tubule showed low expression of Sox9 and UTF1 that was detected out of the main location of the responsible cells for these expressions. Our bioinformatics analysis clearly and strongly indicated the relation between UTF1 in undifferentiated spermatogonia and Sox9 in Sertoli cells mediated by POU5F1.

**Conclusion:**

Generally, this study showed the negative effect of POU5F1 as a mediator between Sertoli cells as the somatic cells within seminiferous tubules and undifferentiated spermatogonia as the spermatogenesis initiator germ cells in non-obstructive conditions.

## Introduction

In mammals, the testis is composed of complex networks of tubes that are functionally unique and are responsible for the expression of male reproductive potential [1]. Germ cells and somatic cells collaborate in testis. Functionally, germ stem cells are responsible for the production of spermatids and then sperms during spermatogenesis stages [2]. Firstly, the spermatogenesis is started by main germ cells, known as spermatogonia (Spg), which are located on the base membrane of seminiferous tubules. Spgs have two fates after the division, first is to renew main germ cells for keeping its pool as progenitor cells and second is to produce primary and secondary spermatocytes [3]. At the final division in the spermatogenesis, secondary spermatocytes become spermatids, which are differentiated to sperms [3].

The normal spermatogenesis needs not only normal germ cells but also an appropriate environment to provide sufficient nutrition and other chemical factors. Sertoli cells are the somatic cells of seminiferous tubules. More research on these cells proved the crucial roles of Sertoli cells in testis through providing normal spermatogenesis and thus are called nurse cells [4].

Sertoli cells have specific morphological features including irregularly-shaped cell membranes, a high number of mitochondria (indicating high metabolic activities), and large nucleus (the size of the nucleus in Sertoli cells depends on the developmental age and the stages of spermatogenesis) [5]. Generally, Sertoli cells mainly take part in the formation of the blood-testis barrier (BTB), the production and differentiation of germ cells, phagocytosis activities, the degeneration of the abnormal sexual cells, the production and secretion of regulatory hormones, the creation of a safe environment for spermatogenesis, and the expression of hormonal receptors in reproductive processes [6–8].

Utf1 is a pluripotency-associated gene and was found to be expressed in embryonic stem (ES) cells, embryonic carcinoma (EC) cells, and primordial germ cells (PGC). Several investigations indicated the UTF1 role in the proliferation stage of ES cells. Furthermore, it was suggested that activation of UTF1 is an important mechanism by which POU5F1 maintains the stem cell state of ES cells [9].

Sox9 is Sry-box containing gene that encodes a transcriptional activator. Sox9 expression is restricted to the Sertoli cell lineage and persists in the adult [10]. Sox9 gene has two independent activity amplifying pathways [8, 11]. First is production of testicular L-PGDS (Lipocalin-Type Prostaglandin D Synthase) leads to the accumulation of PGD2 (Prostaglandin D2), which in turn activates Sox9 transcription and nuclear translocation of SOX9 [12, 13]. This mechanism participates together with FGF9 (fibroblast growth factor 9) as an amplification system of Sox9 gene expression and activity during mammalian testicular organogenesis [14–17].

The main aim of the present investigation was understanding of correlation gene expression pattern between germ cells and somatic cells in non-obstructive azoospermia. Our results would be helpful for further researches with a deep focus on finding treatment for infertility like non-obstructive azoospermia.

## Materials and methods

In this experimental study, we used human non-obstructive samples from the 2 adults male patient’s biopsies. They were obtained from Institute of Anatomy and Cell biology, Medical Faculty, University of Heidelberg. Also, testis samples from 3 pair’s mice (C57BL/6 strain) were obtained from pasture institute and after putting in an enzymatic digestive solution including Dispase (0.5 mg / ml) (Sigma Aldrich), DNAse (0.5 mg / ml) (Sigma Aldrich), and Collagenase (0.5 mg / ml) (Sigma Aldrich) used for further Immunostaining process.

### Immunohistochemical staining

Testis samples were fixed in 4% paraformaldehyde. After cutting of the testis tissue blocks with a microtome (about 10 μm thickness), sections were mounted on slides and stored at room temperature (RT) until used. Before staining, all of the segments were deparaffinized with xylene and rehydrated in an ethanol arrangement. After heat mediated antigen retrieval (10 mM Sodium Citrate Buffer, pH 6 or 1 mM EDTA, pH 8), non-specific binding was blocked with 10% serum and 0.3% Triton in PBS and immunofluorescence staining was performed as explained above (at 95 °C for 20 min).

### PPI network construction and analysis

To construct the PPI network, we used the Search Tool for the STRING version 11.5 (Retrieval of Interacting Genes/Proteins database, https://string-db.org/). STRING is a web database intends to integrate all known and predicted interactions between proteins, including physical interactions and functional associations (Szklarczyk, Gable et al. 2021). STRING app in Cytoscape Software (v 3.8.2) was used to construct the PPI network.

### Gene enrichment analysis

To investigate the functions of the validated genes involved in the sub-network, we have performed the STRING Enrichment analysis in the Cytoscape Software.

## Results

**Fig. 1:**
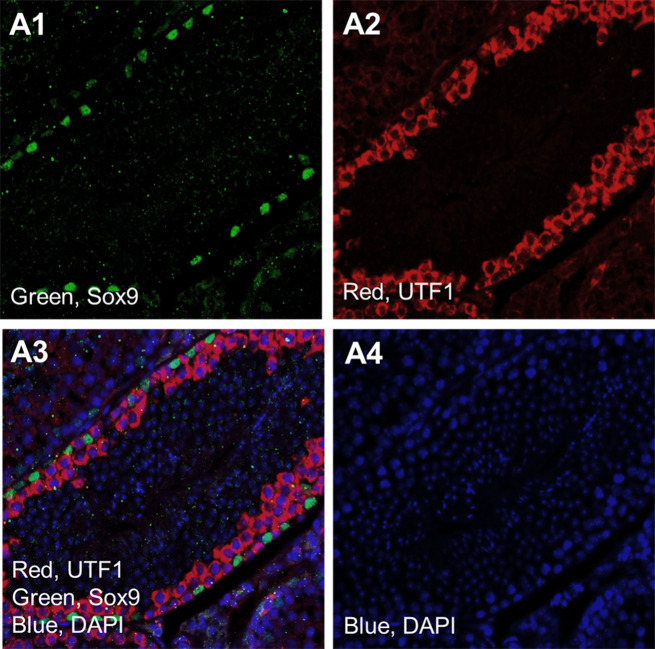
Immunohistochemistry analysis in mice seminiferous tubule as a control pattern. Sox9 expression in the Sertoli cells (**A1**), UTF1 expression (**A2**), Merge image of UTF1 and Sox9 and blue DAPI (**A3**). Blue DAPI for nuclear staining (**A4**). (Scale bar = 50 μm)

**Fig. 2:**
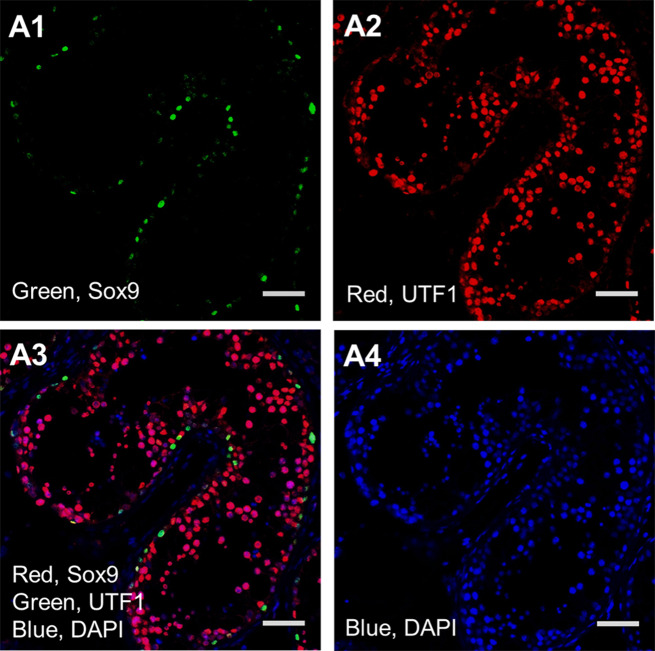
Immunohistochemistry analysis of non-obstructive human seminiferous tubule. Sox9 expression in the Sertoli cells (**A1**), UTF1 expression (**A2**), Merge image of UTF1 and Sox9 and blue DAPI (**A3**). Blue DAPI for nuclear staining (**A4**). (Scale bar = 50 μm)

**Fig. 3:**
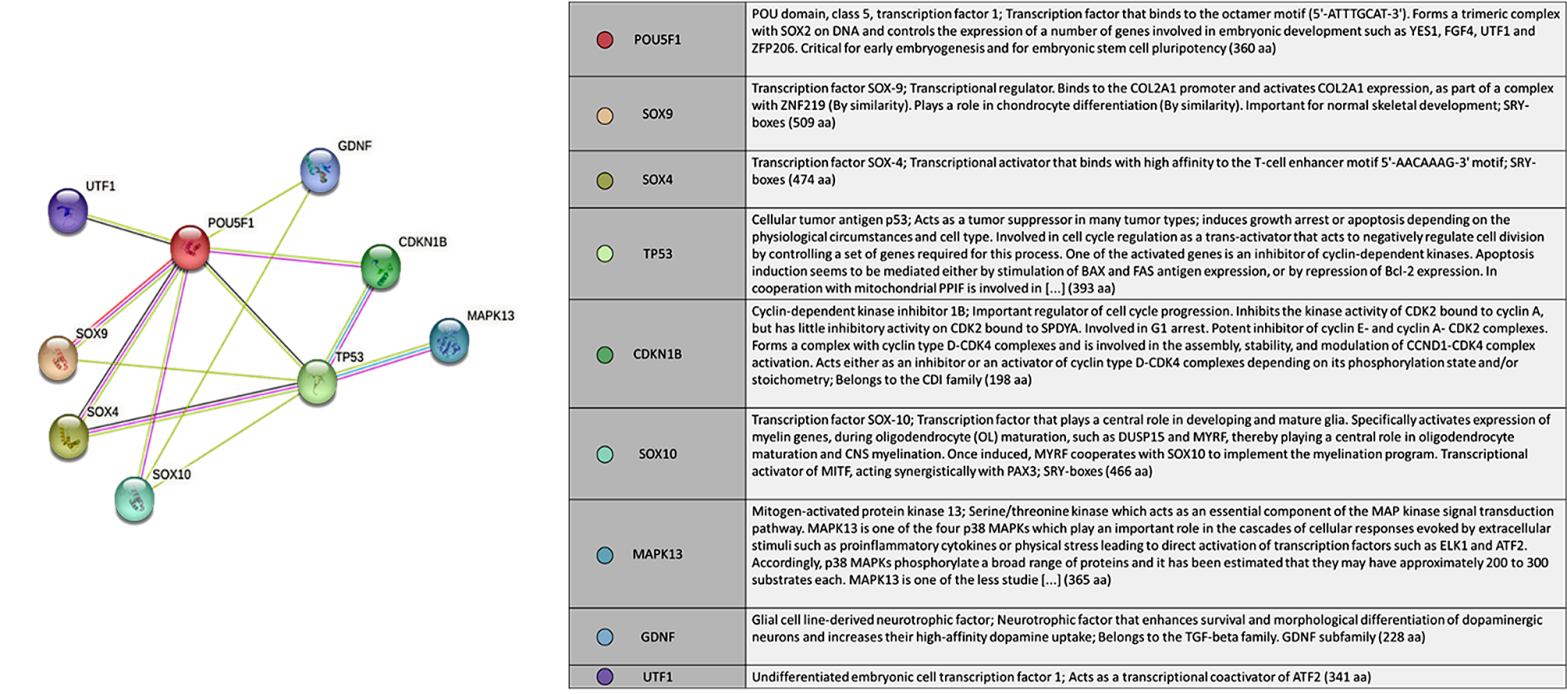
The functional enrichment analysis of selected genes that have direct interaction with UTF1 and Sox9. Different colored parts of the circles refer to the related biological processes and with line thickness being indicative of evidence strength for a predicted interaction

**Fig. 4:**
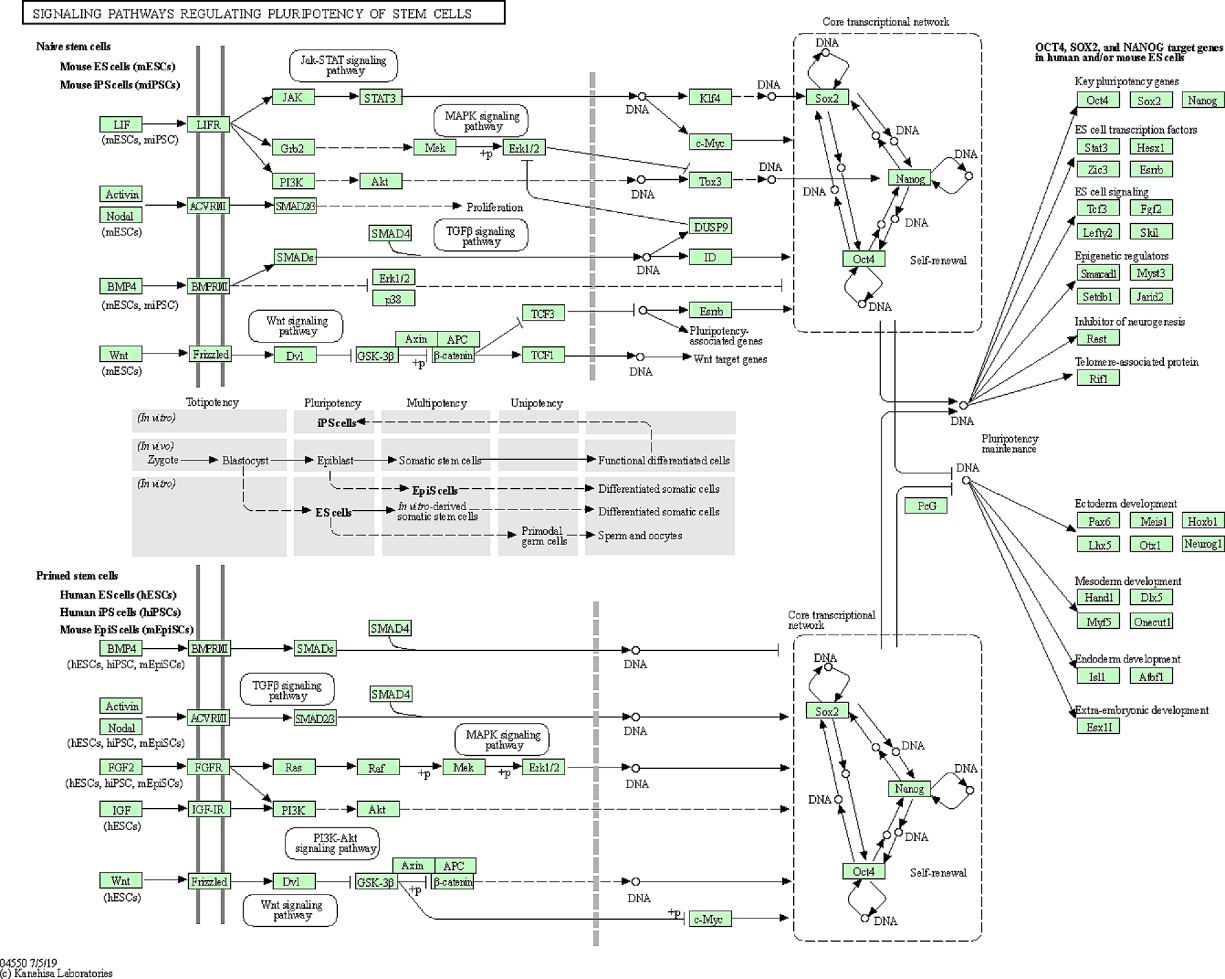
KEGG view on Signaling pathways regulating pluripotency of stem cells

As a control experiment, we used Sox9 and UTF1 expression in mice seminiferous tubules by Immunohistochemistry analysis (Fig. 1). Our analysis revealed the expression of both markers near the basal membrane as we expected based on the specific expression of Sox9 in Sertoli cells (Fig. 1A) and UTF1 in undifferentiated germ cells (Fig. 1B). Confocal scanning UV-laser microscope images proved the expression of UTF1 and sox9 out of the common compartment of seminiferous tubules of non-obstructive azoospermia (Fig. 2). The giant component of the “Stem cell” PPI network generated by STRING and Cytoscape consisted of 100 nodes and 1996 edges (All nodes represent proteins and edges represent protein-protein associations). According to the network, UTF1, Sox9 and POU5F1 are three nodes that we set them first (Fig. 3). We further searched the first neighbors’ nodes with these three genes and we generate a sub-network. This sub-network includes 37 nodes. This suggests that UTF1, Sox9 and POU5F1 are in a highly tight correlation with other stem cell genes. We implemented STRING Enrichment analysis on 14 genes that we had selected, to evaluate molecular functions and cellular locations associated with UTF1, Sox9 and POU5F1 (Fig. 3). We selected some biological processes based on the aim of our experimental studies including; embryo development, stem cell differentiation, and stem cell population maintenance. Also, by TISSUES analysis the localization of genes in pluripotent stem cells, trophectoderm, spermatogonium, adult stem cell, embryoid body, ectoderm, blastocyst, morula, and embryonic stem cell. Furthermore, by KEGG Pathways we identified that if our genes participate in the signaling pathways regulating pluripotency of stem cells. In addition, with COMPARTMENTS analysis we show the localization of our genes in cells (Fig. 4).

## Discussion

In the testicular niche, supportive Sertoli cells and interstitial tissue cells produce various growth factors that stimulate the self-renewal, proliferation, and differentiation of SSCs. Male infertility can arise from factors such as hormonal imbalances, physical challenges, psychological or behavioral issues [18]. Physical problems in the testis, such as a low number or absence of SSCs or supportive cells, can contribute to male infertility [19]. Currently, patients with deficiencies in both germ cells and somatic cells (supportive cells) in the testis face challenges, leading to the differentiation of undifferentiated SSCs into sperm.

Sertoli cells play a crucial role in regulating spermatogonial cell functions during spermatogenesis in the male testes [20]. The Sox gene family, particularly Sox9, serves as a transcription factor critical for cellular differentiation during embryogenesis in various tissues, including Sertoli cells of the testis [21], neural crest cells, and chondrocytes [22, 23]. Since Sox9 expression is limited to Sertoli cells and is used as a specific marker for their identification in the testis, we utilized Sox9 as a Sertoli-specific marker in this study [24, 25]. It is known that Sox9 also plays a crucial role in vertebrate sex determination, regulated by the SRY gene [22]. Studies have shown that the pattern of Sox9 expression in Sertoli cells differs significantly in patients with non-obstructive azoospermia or Sertoli cells only syndrome (SCOS). Kuo-Chung Lan et al. reported up-regulation of Sox9 protein expression and a different expression pattern in patients with SCOS [23]. Given the critical roles of Sertoli cells in male reproduction determination, several investigations have focused on the chemical and physiological features of Sertoli cells to understand their exact mechanisms in supporting spermatogenesis [26–31].

On the other hand, UTF1 is a specific marker for the undifferentiated compartment and was utilized in the human non-obstructive azoospermia testis in this study. Immunohistochemistry results indicated a reduction in Sox9 and UTF1 expression in human non-obstructive testes, clearly outside the main Sertoli and undifferentiated cells’ niche. Our bioinformatics analysis and signaling pathway investigation revealed the impact of POU5F1 on UTF1 expression in undifferentiated spermatogonia and demonstrated a significant connection with Sox9 expression. This might explain the reduction of these genes and proteins in non-obstructive azoospermia testes.

## Conclusion

In this project we confirmed that there is correlation between the POU5F1 and regulation of the Sox9 (Sertoli-specific marker) and UTF1 (undifferentiated germ cell marker). Our results from immunohistochemistry of non-obstructive azoospermia revealed a low expression of sox9 and UTF1 in the locations that were not related to the Sertoli cells and undifferentiated spermatogonia respectively. Our bioinformatics analysis indicated a mediatory role of POU5F1 between UTF1 and Sox9. The presented informations will be useful for further advanced studies against infertility.

## Data Availability

The data sets analyzed for the current study are available from the corresponding author on reasonable request.

## References

[1] Parekh PA, Garcia TX, Hofmann M-C (2019). Regulation of GDNF expression in sertoli cells. Reproduction.

[2] Lee S (2019). Differential Regulation of TLE3 in sertoli cells of the testes during postnatal development. Cells.

[3] Azizi H et al. *Investigation of VASA Gene and protein expression in neonate and adult testicular germ cells in mice in vivo and in Vitro*. Cell J (Yakhteh), 2020. 22(2). 10.22074/cellj.2020.6619 PMC6874794 31721531

[4] Pelusi C (2020). Effect of clomiphene citrate treatment on the sertoli cells of dysmetabolic obese men with low testosterone levels. Clin Endocrinol.

[5] Paduch DA et al. *Aberrant gene expression by sertoli cells in infertile men with sertoli cell-only syndrome*. PLoS ONE, 2019. 14(5). 10.1371/journal.pone.0216586 PMC6508736 31071133

[6] Miller SR et al. *Cell model for studying Nucleoside transporters, a key component of the blood-testis barrier*. FASEB J, 2019. 33(1_supplement): p. 507.12-507.12.

[7] Hui L (2020). Matrix metalloproteinase 9 facilitates Zika virus invasion of the testis by modulating the integrity of the blood-testis barrier. PLoS Pathog.

[8] Tao S (2019). Adverse effects of bisphenol A on sertoli cell blood-testis barrier in rare minnow Gobiocypris rarus. Ecotoxicol Environ Saf.

[9] Su L, et al. Testin regulates the blood-testis barrier via disturbing occludin/ZO‐1 association and actin organization. Journal of Cellular Physiology; 2020. 10.1002/jcp.29541 31975378

[10] Bernardino RL (2019). Carbonic anhydrases are involved in mitochondrial biogenesis and control the production of lactate by human sertoli cells. FEBS J.

[11] Ni F-D, Hao S-L, Yang W-X (2019). Multiple signaling pathways in sertoli cells: recent findings in spermatogenesis. Cell Death Dis.

[12] Nayernia K (2006). In vitro-differentiated embryonic stem cells give rise to male gametes that can generate offspring mice. Dev Cell.

[13] Nayernia K (2006). Derivation of male germ cells from bone marrow stem cells. Lab Invest.

[14] Monsefi M (2013). Mesenchymal stem cells repair germinal cells of seminiferous tubules of sterile rats. Iran J Reprod Med.

[15] Cakici C (2013). Recovery of fertility in azoospermia rats after injection of adipose-tissue-derived mesenchymal stem cells: the sperm generation. Biomed Res Int.

[16] Yazawa T (2006). Differentiation of adult stem cells derived from bone marrow stroma into Leydig or adrenocortical cells. Endocrinology.

[17] Bucay N (2009). A novel approach for the derivation of putative primordial germ cells and sertoli cells from human embryonic stem cells. Stem Cells.

[18] Azizi H, Hamidabadi HG, Skutella T (2019). Differential proliferation effects after short-term cultivation of mouse spermatogonial stem cells on different feeder layers. Cell J (Yakhteh).

[19] Bordeaux J (2010). Antibody validation. Biotechniques.

[20] Khuder HA-Q (2021). Androgen hormone and male infertility. Int J Res Appl Sci Biotechnol.

[21] Ibtisham F, Honaramooz A (2020). Spermatogonial stem cells for in vitro spermatogenesis and in vivo restoration of fertility. Cells.

[22] Akiyama H et al. *The transcription factor Sox9 has essential roles in successive steps of the chondrocytes differentiation pathway and is required for expression of Sox5 and Sox6* Genes & development, 2002. 16(21): p. 2813-28. 10.1101/gad.1017802 PMC187468 12414734

[23] Bi W (2001). Haploinsufficiency of Sox9 results in defective cartilage primordia and premature skeletal mineralization. Proc Natl Acad Sci USA.

[24] Angelozzi M, Lefebvre V. SOXopathies: growing family of developmental disorders due to SOX mutations. Trends in Genetics; 2019. 10.1016/j.tig.2019.06.003 PMC6956857 31288943

[25] Rehman ZU, et al. Expression Profile of Sox5 and Sox6 in sertoli and Spermatogonial cells in growing mice Testis. Volume 26. KAFKAS ÜNİVERSİTESİ VETERİNER FAKÜLTESİ DERGİSİ; 2020. 1.

[26] Hemendinger RA (2002). Identification of a specific sertoli cell marker, Sox9, for use in transplantation. Cell Transplant.

[27] Kent J (1996). A male-specific role for SOX9 in vertebrate sex determination. Development.

[28] Lan KC (2013). Up-regulation of SOX9 in sertoli cells from testiculopathic patients accounts for increasing anti-mullerian hormone expression via impaired androgen receptor signaling. PLoS ONE.

[29] Franke WW, Grund C, Schmid E (1979). Intermediate-sized filaments present in sertoli cells are of the vimentin type. Eur J Cell Biol.

[30] Virtanen I (1986). Peritubular myoid cells of human and rat testis are smooth muscle cells that contain desmin-type intermediate filaments. Anat Rec.

[31] Bilinska B (1989). Visualization of the cytoskeleton in Leydig cells in vitro. Effect of luteinizing hormone and cytoskeletal disrupting drugs. Histochemistry.

